# Dual function antibody targeting αvβ3 and PD-L1 provide a promising strategy for solid tumor therapy

**DOI:** 10.3389/fimmu.2025.1691774

**Published:** 2026-01-12

**Authors:** Guixia Li, Wenlei Li, Liuli Wang, Yuxin Bi, Yibo Wang, Xuemin Zheng, Ruijia Hao, Yanchen Yin, Yan Yu, Huaibin Mu, Jian Li, Xiaohui Ma, Shuiping Zhou, Jin Han, Genbei Wang, Ruijing Huang

**Affiliations:** 1 Pharmacology and Toxicology Research Department, Tasly Research Center, Tasly Pharmaceutical Group Co., Ltd., Tianjin, China; 2 Biologics Development Department, Tasly Research Center, Tasly Pharmaceutical Group Co., Ltd., Tianjin, China; 3 Tasly Biopharmaceuticals Co., Ltd., Tianjin, China

**Keywords:** antibody, PD-L1, solid tumor, tumor immunology, αvβ3

## Abstract

**Background:**

The inhibition of the PD-1/PD-L1 axis has exhibited significant advancements in cancer immunotherapy, improving patient outcomes in various cancers. However, the clinical efficacy of these monotherapies remains limited in many cases. Integrin αvβ3 has been identified as a positive regulator of PD-L1 expression and a critical contributor to cancer immune evasion. To address this, we developed a dual function antibody, B1451, that recognizes both PD-L1 and αvβ3 and evaluated its antitumor efficacy in pre-clinical models *in vitro* and *in vivo*.

**Methods:**

We first analyzed the correlation between PD-L1 and αvβ3 expression, as well as the role of αvβ3 in modulating sensitivity to immunotherapy, using the TISIDB database. Subsequently, we designed and constructed a dual function PD-L1×αvβ3 antibody (B1451) by conjugating an integrin αvβ3-binding peptide to the C-terminal of the heavy chain of the anti-PD-L1 monoclonal antibody, Atezolizumab, using a (G4S)×3 linker. The antitumor efficacy of B1451 was then evaluated in preclinical models *in vitro* and *in vivo*.

**Results:**

Our findings demonstrated a significant positive correlation between the gene expression of PD-L1 and αvβ3 across various human solid tumors. Additionally, high αvβ3 expression appears to influence the sensitivity to immunotherapy. The dual function antibody B1451 was capable of recognizing human PD-L1 and αvβ3 antigens, effectively blocking both the PD-1/PD-L1 and vitronectin/αvβ3 pathways. B1451 inhibited tumor cell migration, adhesion, and angiogenesis *in vitro*, and exhibited superior anti-tumor activity *in vivo* than monotherapy.

**Conclusion:**

The dual function antibody targeting both αvβ3 and PD-L1 holds the potential to reverse immune evasion and exhibit synergistic anti-tumor effects, offering a promising therapeutic strategy for the treatment of solid tumor.

## Introduction

Cancer remains one of the most significant global health challenges. Over the past few years, cancer immunotherapy has achieved remarkable clinical breakthroughs, with particular success in targeting the PD-1/PD-L1 axis. This approach represents a cutting-edge treatment for various cancer types and has significantly advanced patient care (1). However, despite substantial progress in inhibiting the PD-1/PD-L1 pathway, the clinical response rates to these monotherapies remain limited, ranging from approximately 10% to 30%. Furthermore, a significant proportion of initial responders eventually develop acquired resistance. Additionally, PD-1/PD-L1 inhibitors often elicit adverse effects due to their lack of specificity for tumor-associated lymphocytes (2–4). As such, developing drugs that target additional tumor-enrichd receptors to enhance immune infiltration into the tumor microenvironment presents a promising strategy to mitigate these challenges and improve patient outcomes. Currently, several dual inhibitors targeting PD-L1 in combination with other tumor-specific molecules are undergoing clinical development (5–7).

Integrins, a superfamily of heterodimeric adhesion receptors composed of α and β subunits, are closely associated with key intracellular signals involved in tumor growth, malignant metastasis, angiogenesis, tumor stem cell maintenance, and immune escape (8–10). Among these, integrin alphav beta3 (αvβ3) is highly expressed in several solid cancers, including melanoma, breast, pancreatic, lung, glioblastoma, high-grade serous ovarian cancer, colorectal cancer, and prostate cancer. Its overexpression is strongly correlated poor prognosis and reduced survival rates (11–16). As one of the most extensively studied integrins in cancer research, integrin αvβ3 has demonstrated a pivotal role in tumor progression. Emerging evidence suggests that αvβ3 regulates tumor cells adhesion and migration by directly interacting with the extracellular matrix (ECM) or modulating the expression of matrix metalloproteinases (MMPs) through the PI3K signaling pathway (12, 17). Furthermore, integrin αvβ3 mediates resistance to EGFR by activating the Galectin-3/KRAS/RalB/TBK1/NF-κB signaling pathway (18). Notably, recent studies highlight the role of integrin αvβ3 in promoting immune evasion by upregulating PD-L1 expression. In gastric cancer, αvβ3 enhances immune evasion and resistance to PD-1 immunotherapy by activating the PI3K/AKT/mTORC2 pathway. Similarly, in other solid tumors, αvβ3 has been shown to regulate PD-L1 expression and contribute to immune system evasion (19–21). Despite several drugs targeting αvβ3 progressing to clinical development, such as cilengitide, their efficacy as monotherapy has been limited in clinical trials (22–26). However, αvβ3 holds significant promise as a target in combination therapies. Preclinical studies have demonstrated that combining anti-PD-1 antibodies with β3-integrin depletion delivers durable therapeutic effects and enhances abscopal immune responses (20). Additionally, combining αvβ3 inhibitors with anti-PD-L1 antibodies has been shown to improve overall survival and achieve long-term tumor control in mouse models (27, 28). Another rapidly emerging approach in cancer treatment, is the use of bispecific antibody (BsAb), which simultaneously target two distinct molecules and have garnered significant attention for their therapeutic potential (29). Therefore, targeting both αvβ3 and the PD-1/PD-L1 axis represents a highly promising therapeutic strategy for the treatment of solid tumors. Nevertheless, despite ongoing research, there are currently no drugs targeting both αvβ3 and PD-L1 that have progressed to the clinical stage.

In this study, we generated a dual function antibody, B1451, designed to simultaneously target both PD-L1 and αvβ3. This was achieved by conjugating an integrin αvβ3 binding peptide to the C-terminal of the heavy chain of the anti-PD-L1 monoclonal antibody, Atezolizumab, via a (G4S)×3 linker (30). The antitumor efficacy of B1451 was evaluated in preclinical models *in vitro* and *in vivo*. Our results revealed that B1451 is capable of reversing immune evasion, inhibiting tumor metastasis, improving the tumor microenvironment, and exhibiting synergistic anti-tumor effects through simultaneous targeting of PD-L1 and αvβ3.

## Results

### Correlations between expression of avβ3 and PD-L1 across human cancers

**Figure 1:**
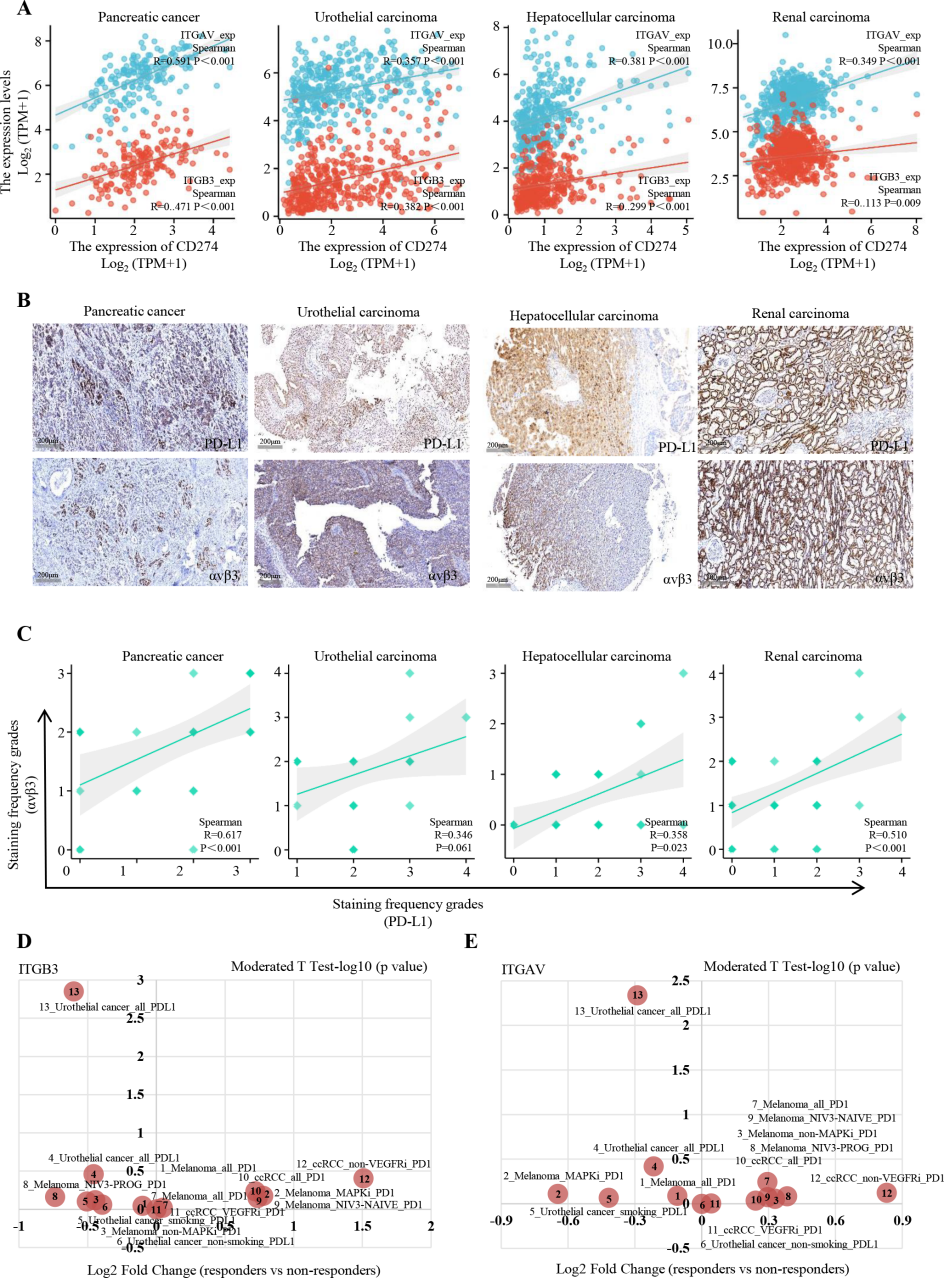
Correlations between expression of avβ3 and PD-L1 across human cancers. **(A)** Correlation analysis of PD-L1 (CD274) with ITGAV or ITGB3 across various cancers. **(B)** IHC staining of PD-L1 and avβ3 in tumors samples, with representative images shown. Scale bar, 200µm. **(C)** Correlation analysis of PD-L1 (CD274) with ITGAV and ITGB3 based on IHC staining data from tumor samples. **(D, E)** Comparison of ITGAV and ITGB3 differences between immunotherapy responders and non-responders across multiple datasets.

Analysis using the Xiantao online tools revealed that the CD274 (PD-L1) gene exhibits high expression levels in tumor samples from hepatocellular carcinoma, pancreatic cancer, renal cancer, and urothelial cancer, alongside elevated expression of αv (ITGAV) and β3 (ITGB3) genes (**Figure 1A**). These findings demonstrate a significant positive correlation between CD274 gene expression and the expression of ITGAV and ITGB3 genes.

Subsequent immunohistochemistry staining further validated these results, showing that high levels of PD-L1 protein (staining frequency of grade 2 or higher) were observed in various cancer types: hepatocellular carcinoma (52.5%, 21/40), urothelial carcinoma (86.7%, 26/30), renal carcinoma (52.0%, 26/50), and pancreatic cancer (66.7%, 20/30). Correspondingly, αvβ3 protein expression was detected in 7.5% (3/40) of hepatocellular carcinoma cases, 73.3% (22/30) of urothelial carcinoma cases, 50.0% (25/50) of renal carcinoma cases, and 73.3% (22/30) of pancreatic cancer cases (**Figures 1B, C** and online **Supplementary Table S1**).

Furthermore, we analyzed the relationship between αvβ3 expression between immunotherapy response using TISIDB database (http://cis.hku.hk/TISIDB). The analysis identified a significant negative correlation between the expression of ITGAV and ITGB3 genes and response to anti-PD-L1 therapy in urothelial cancer. Specifically, the correlation coefficients were as follows: ITGAV (r=-0.289, p=0.0046) and ITGB3 (r=-0.602, p=0.00141) (**Figures 1D, E**).

These results suggest that αvβ3 may influence sensitivity to immunotherapy and indicate its potential as a strong modulator of anti-tumor effects when combined with PD-L1 inhibitors in the treatment of solid tumor therapy.

### Characterization of the binding capability of anti-PD-L1×avβ3 dual function antibody (B1451)

**Figure 2:**
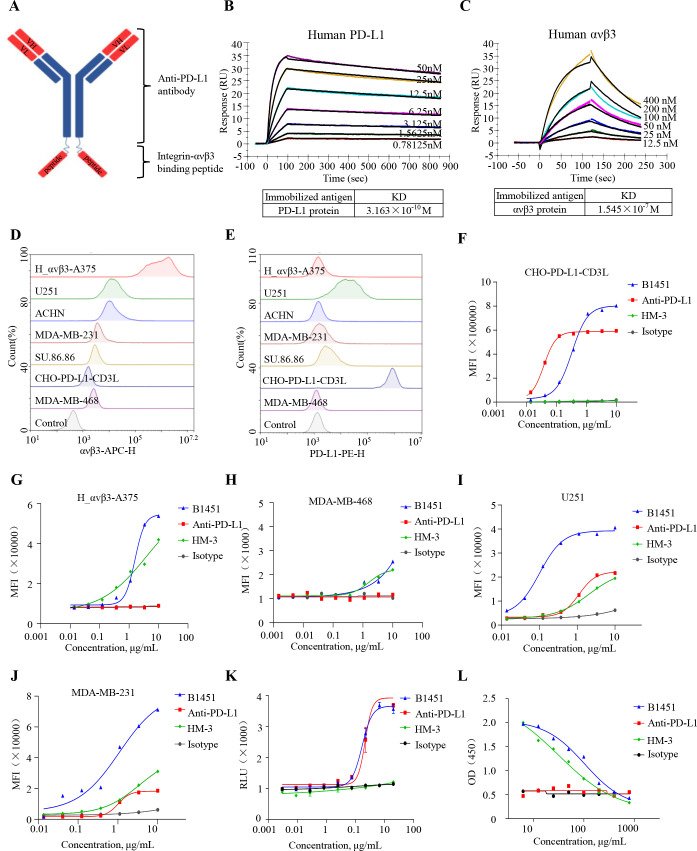
Characterization of anti-PD-L1×avβ3 dual function antibody (B1451). **(A)** Schematic representation of B1451, constructed by conjugating HM-3, an RGD-modified peptide targeing αvβ3, to the C-terminus of the heavy chain of the anti-PD-L1 monoclonal antibody Atezolizumab via a (G4S)×3 linker. **(B, C)** Surface plasmon resonance analysis confirmed the binding of B1451 to the targets PD-L1 **(B)** and avβ3 **(C)** at the indicated concentrations. **(D, E)** Expression of PD-L1 and avβ3 in tested cell lines was determined by flow cytometry. Histograms show the median fluorescence intensity (MFI) of avβ3 **(D)** and PD-L1 **(E)**. **(F–J)** Flow cytometry analysis of the binding of increasing concentrations of B1451 to CHO-PD-L1-CD3L pre-blocked avβ3 antigen **(F)**, H_avβ3 A375 pre-blocked PD-L1 antigen **(G)**, MDA-MB-468 **(H)**, U251 **(I)** and MDA-MB-231 **(J)** cell lines. The blocking effect of B1451 on the PD-1/PD-L1 pathway was assessed by co-culturing CHO-PD-L1-CD3L cells with Jurkat-PD-1-NFAT reporter cells **(K)**. The blocking effect of B1451 on the αvβ3/vitronectin pathway was evaluated by ELISA at the indicated concentrations **(L)**.

The anti-PD-L1×avβ3 dual function antibody (referred as B1451) was engineered by conjugating HM-3, an RGD-modified peptide targeing αvβ3 (30), to the C-terminus of the heavy chain of the anti-PD-L1 monoclonal antibody, Atezolizumab, via a (G4S)×3 linker (**Figure 2A**). Surface plasmon resonance (SPR) binding affinity analysis revealed that B1451 interacts with avβ3, exhibiting a calculated KD of 15.45 μM. In contrast, it demonstrated a significantly stronger binding affinity for the PD-L1 protein, with a calculated KD of 31.63 nM (**Figures 2B, C**). Next, we assessed the surface expression of PD-L1 and avβ3 on CHO-PD-L1-CD3L, SU.86.86, ACHN, U251, H_avβ3 A375, MDA-MB-231, and MDA-MB-468 cell lines. We observed that except for MD-MB-468 cells, both positive expression of PD-L1 and avβ3 on the surfaces of all the other tested cell lines (**Figures 2D, E**). Then, we adopted a pre-blocking strategy of one antigen to study the binding specificity of B1451 on CHO-PD-L1-CD3L and H_avβ3 A375 cells. Additionally, we selected MDA-MB-231 and U251 cells, which co-express both PD-L1 and avβ3, to evaluate the binding activity of B1451. Then, we selected MDA-MB-468 cells, which exclusively weak expression of avβ3, to assess the binding activity of B1451. The results showed that the binding activity of B1451 to CHO-PD-L1-CD3L cells was comparable to that of anti-PD-L1 after avβ3 was blocked using HM-3 (**Figure 2F**). Similarly, on H_avβ3 A375 cell, B1451 and HM-3 showed comparable binding after PD-L1 antigen was blocked using anti-PD-L1 (**Figure 2G**). However, only weak binding was observed of B1451 and HM-3 on MDA-MB-468 cells, which is consistent with the target expression results (**Figure 2H**). However, the binding ability of B1451 was significantly stronger than that of anti-PD-L1 or HM-3 alone on U251 and MDA-MB-231 cells (**Figures 2I, J**). The results confirmed the functional specificity of B1451. Moreover, the binding ability of B1451 may has an advantage to dual-target cells.

Additionally, it is well established that the binding of PD-1 to PD-L1 inhibits T cell activation. In this study, the PD-L1 blocking ability of B1451 and the parental antibody was assessed using a PD-1/PD-L1 blockade bioassay. The luminescence signal of the downstream NFAT reporter was measured to evaluate the blocking effect. The results demonstrated that B1451 effectively activated NFAT by disrupting the interaction between PD-1 and PD-L1 (**Figure 2K**), with a comparable effect to anti-PD-L1. Vitronectin, a primary ligand of αvβ3, specifically binds to the arginine-glycine-aspartic acid (RGD) sequence. To evaluate the avβ3 blocking ability of B1451 and the parental antibody, an avβ3/vitronectin blockade bioassay was performed. As expected, B1451 and HM-3 efficiently inhibited avβ3/vitronectin signaling, whereas anti-PD-L1 showed minimal blocking effect (**Figure 2L**).

Collectively, these findings demonstrated that the PD-L1×avβ3 dual function antibody B1451 is capable of recognizing and binding to cells co-expressing PD-L1 and avβ3, with comparable blocking activity of PD-1/PD-L1 and avβ3/vitronectin to parent antibody.

### B1451 induces tumor cell migration, invasion, angiogenesis, and tumor cell appoptosis

**Figure 3:**
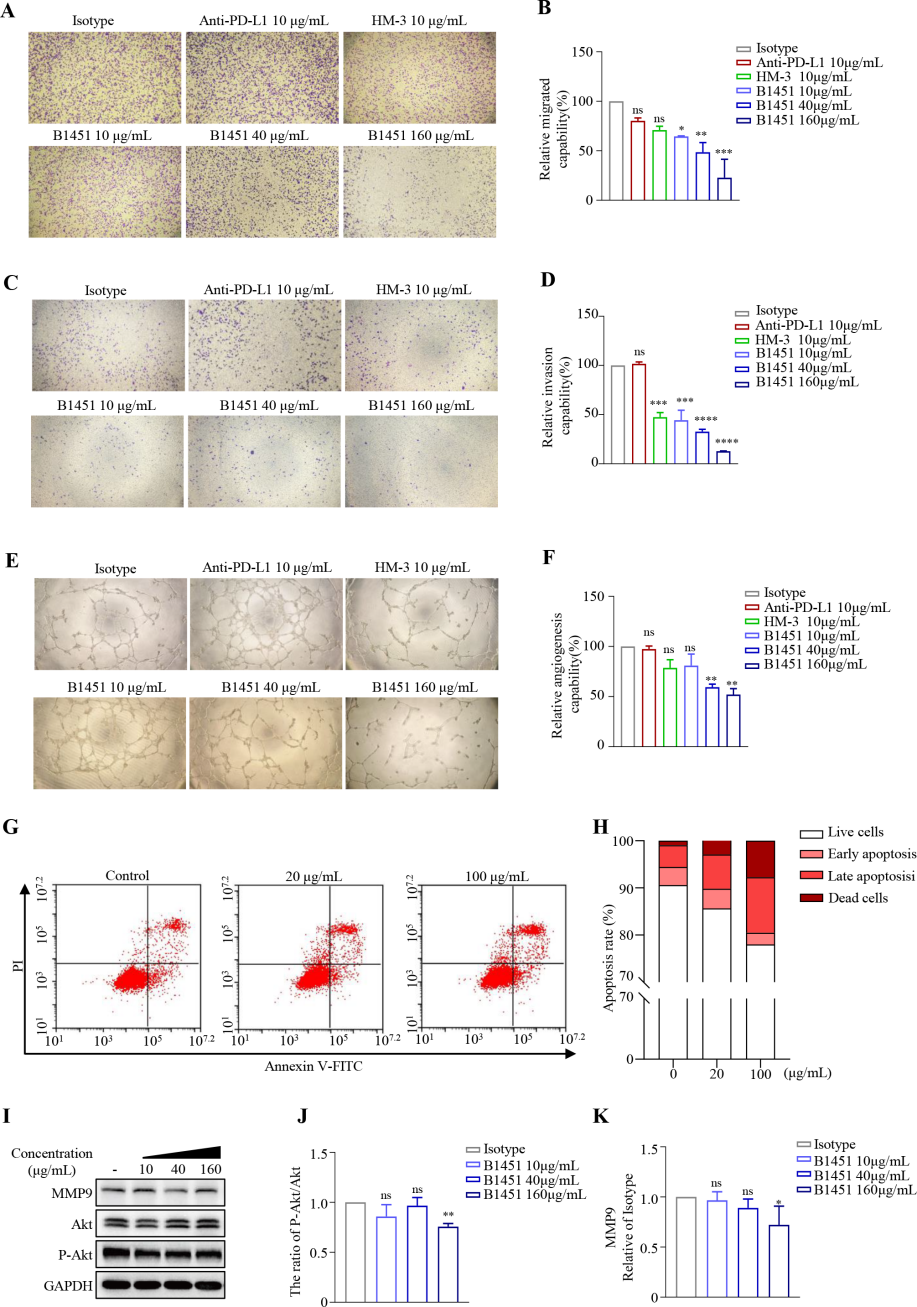
B1451 inhibits tumor cell migration, invasion and angiogenesis. **(A–D)** The migration and invasion abilities of H_αvβ3 A375 cells were evaluated using a transwell assay and compared with the control group. Representative images are shown for each condition. **(E, F)** Tube formation assays were performed in HUVEC cells to evaluate the impact of B1451 on angiogenesis. Representative images are shown for each condition. **(G)** Dot plot analysis of flow cytometry of apoptotic H_αvβ3 A375 cells treated with or without the B1451 antibody. **(H)** Apoptotic cells were quantified and categorized into early and late apoptotic cells. **(I–K)** Levels of AKT phosphorylation and MMP9 were analyzed by immunoblot. Statistical significance was analyzed using ANOVA test. ^*^p<0.05; ^**^p<0.01; ^***^p<0.001; ^****^p<0.0001, ns, not significant.

We further investigated the effects of B1451 on tumor cell migration and invasion using H_αvβ3 A375 cells, as well as its impact on tube formation in HUVEC cells. The results showed that B1451 exerted a marked inhibitory effect on tumor cell migration and invasion in a dose-dependent manner, with concentrations ranging from 10-160 μg/mL. The effect of B1451 on inhibiting migration and invasion of H_αvβ3 A375 cells was comparable to that of an equal dose of HM-3, while no inhibitory effect was observed in the anti-PD-L1 treatment group (**Figures 3A–D**). Moreover, B1451 and HM-3 significantly impaired tube formation in HUVEC cells (**Figures 3E, F**).

The antitumor activity of B1451 was also evaluated *in vitro* using Annexin V-FITC/PI staining to measure apoptotic cell death in H_αvβ3 A375 cells. The results revealed dose-dependent induction of apoptosis by B1451 (**Figures 3G, H**).

We also have carried out immunoblot analysis to detect the phosphorylation level of AKT (a key molecule in the downstream signaling pathway) and the expression of MMP9 protein. The results showed that B1451 downregulated AKT phosphorylation and MMP9 protein expression in a concentration-dependent manner (**Figures 3I–K**).

Together, these findings suggest that B1451 may exert an anti-tumor metastasis effect by blocking αvβ3, downregulating the downstream PI3K signaling pathway, to induce inhibiting tumor cell migration, adhesion, and angiogenesis. Furthermore, B1451 exerts antitumor effect by promoting apoptosis of tumor cells.

### B1451 displays stronger anti-tumor effect in mouse models

To evaluate the anti-tumor efficacy of B1451 *in vivo*, multiple mouse tumor models were established.

**Figure 4:**
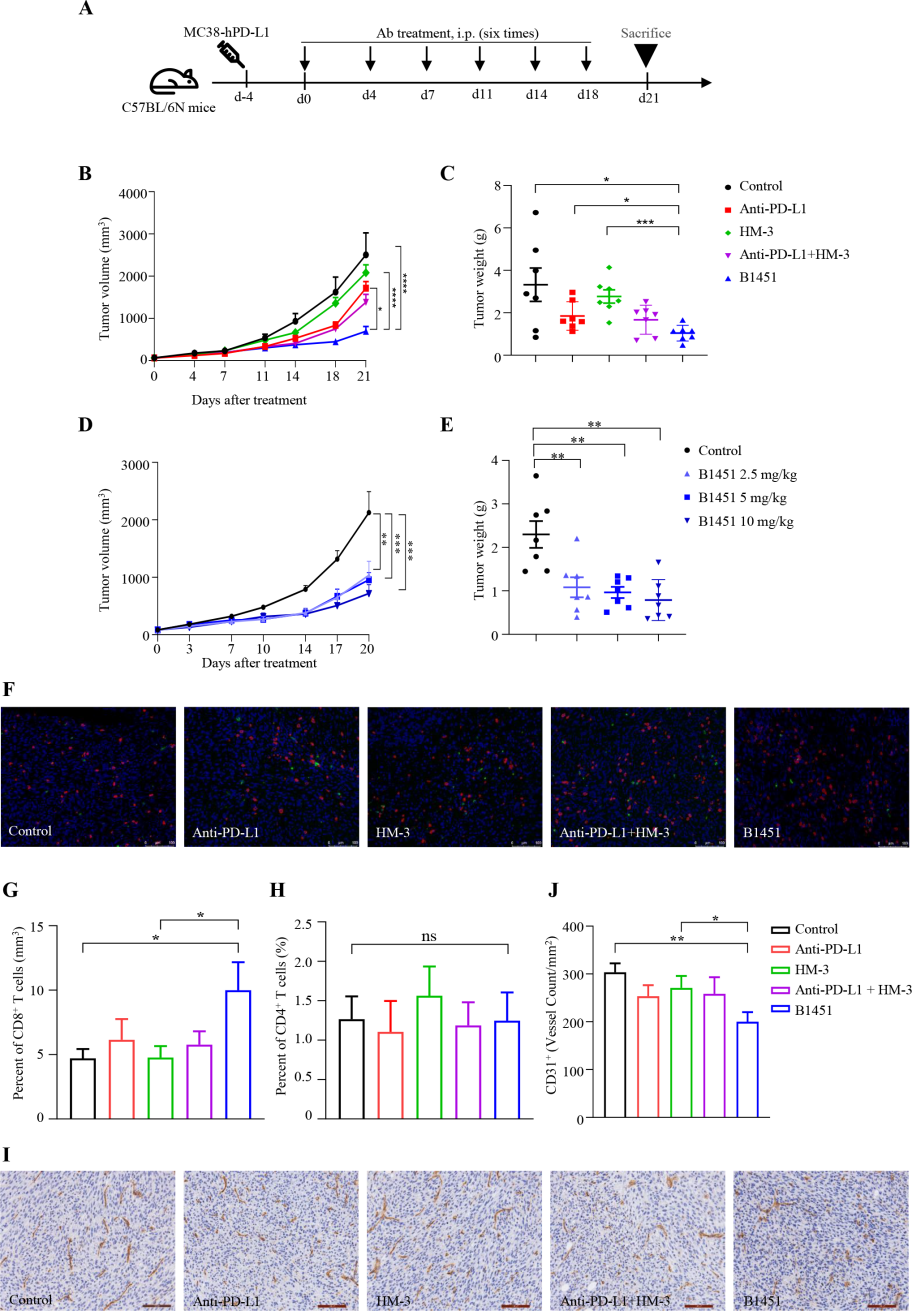
B1451 exhibits stronger anti-tumor effects in MC38-hPD-L1 xenograft mouse models. **(A)** Schematic representation of the MC38-hPD-L1 xenograft mouse model. C57BL/6N mice were subcutaneously injected with 1×10^6^ MC38-hPD-L1 cells. Four days later, the animals will be randomly grouped to start administration. **(B, C)** MC38-hPD-L1 bearing mice treated with B1451 (2.5 mg/kg), anti-PD-L1 (2.5 mg/kg), HM-3 (0.97 mg/kg), or anti-PD-L1 (2.5 mg/kg) combination with HM-3 (0.97 mg/kg) twice a week for three consecutive weeks (n=7 per group). Tumor volume **(B)** and tumor weight **(C)** were assessed. **(D, E)** MC38-hPD-L1 bearing mice treated with varying doses of B1451 (2.5, 5, 10 mg/kg) twice a week for three consecutive weeks (n=7 per group). Tumor volume **(D)** and tumor weight **(E)** were evaluated. **(F-H)** Representative multiplex immunohistochemistry images depicting immune cell infiltration **(F)**, with quantification of the percentages of CD8^+^**(G)** and CD4^+^**(H)** T cells among all tissues cells performed manually. **(I, J)** Immunohistochemistry was performed to assess the expression levels of CD31^+^ in mouse tumor tissues. Statistical significance was analyzed using ANOVA test and t tests. ^*^p<0.05; ^**^p<0.01; ^***^p<0.001; ^****^p<0.0001, ns, not significant.

**Figure 5:**
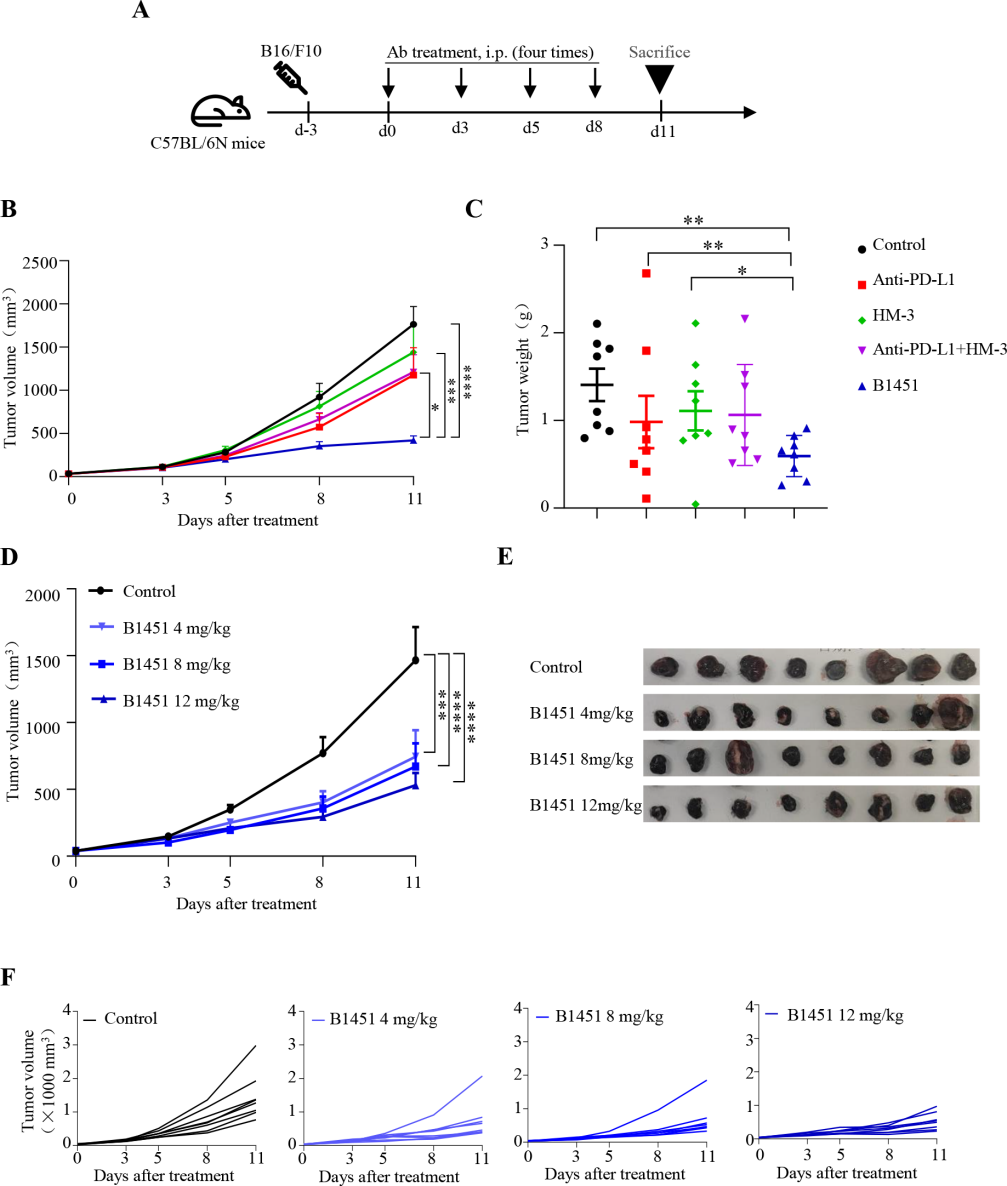
B1451 exhibits stronger anti-tumor effects in B16/F10 xenograft mouse model. **(A)** Schematic representation of the B16/F10 xenograft mouse model. C57BL/6N mice were subcutaneously injected with 5×10^5^ B16/F10 cells. Three days later, the animals will be randomly grouped to start administration. **(B, C)** Tumor-bearing mice were randomly assigned to five groups and treated with with B1451 (4 mg/kg), anti-PD-L1 (4 mg/kg), HM-3 (1.6 mg/kg), or anti-PD-L1 (4 mg/kg) combination with HM-3 (1.6 mg/kg) for four times weeks (n=8). Tumor volume **(B)** and tumor weight **(C)** of all mice were shown. **(D–F)** B16/F10 bearing mice were treated with B1451 (4, 8, 12 mg/kg) for four times (n=8). Tumor volume **(D)**, tumor photo **(E)**, and individual tumor growth curve **(F)** of all mice are shown. Statistical significance was analyzed using ANOVA test and t tests. ^*^p<0.05; ^**^p<0.01; ^***^p<0.001; ^****^p<0.0001.

First, we assessed the anti-tumor activity of B1451 in a MC38-hPD-L1 xenograft model. Tumor-bearing mice were treated with B1451, anti-PD-L1, HM-3, or a combination of anti-PD-L1 and HM-3, and tumor progression was monitored (**Figure 4A**). Monotherapy with anti-PD-L1 and HM-3 demonstrated weaker anti-tumor effects, whereas treatment with B1451 and the combination of anti-PD-L1 and HM-3 exhibited strong anti-tumor efficacy (n=7, **Figures 4B, C** and online **Supplementary Figure S1A**). Notably, treatment with B1451 resulted in significantly stronger anti-tumor effects compared to individual treatments with either anti-PD-L1 or HM-3 alone. These findings were further validated using B16/F10 xenograft model and Renca xenograft model, where tumor progression under B1451 treatment echoed the aforementioned results (**Figures 5A–C** and online **Supplementary Figure S2**).

Next, we examined whether the therapeutic efficacy of B1451 was dose dependent. In the MC38-hPD-L1 xenograft model, mice were treated with B1451 at doses ranging from 2.5 to 10 mg/kg. The results demonstrated a clear dose-dependent increase in B1451’s *in vivo* anti-tumor activity (n=7, **Figures 4D, E** and online **Supplementary Figure S1B**). Similarly, dose-dependent efficacy was confirmed using the B16/F10 xenograft model, where mice received B1451 doses ranging from 4 to 12 mg/kg (n=8, **Figures 5D–F**). Additional dose-dependent anti-tumor efficacy was observed in the H22 xenograft model, further confirming the potency of B1451 (online **Supplementary Figure S3**).

Moreover, we explored possible mechanisms underlying the enhanced anti-tumor efficacy of B1451 in the MC38-hPD-L1 tumor model using multiplex immunofluorescence and immunohistochemical analyses. Multiplex immunofluorescence revealed a significant increase in CD8^+^ T cell infiltration in tumors treated with B1451 compared to vehicle control or single agent treatment (**Figures 4F, G**), While no significant differences were observed in CD4^+^ T cell infiltration (**Figure 4H**). Moreover, it can be observed that B1451 treatment can reduce the expression of PD-L1 in tumor tissues, thereby alleviating immune escape (online **Supplementary Figure S4**). Immunohistochemical analysis further showed a significant reduction in CD31^+^ staining in tumors treated with B1451, indicating a decrease in intratumoral microvessel density (**Figures 4I, J**).

Overall, these findings demonstrate that B1451 induces remarkable anti-tumor activity *in vivo*, which surpasses the efficacy of either anti-PD-L1 or HM-3 monotherapy. The enhanced tumor growth suppression mechanism may involve increased infiltration of cytotoxic CD8^+^ T cells, reduced PD-L1 expression to alleviate immune suppression, and suppression of intratumoral angiogenesis through reduced microvessel density.

### B1451 induces tumor regression in humanized mice *in vivo*

**Figure 6:**
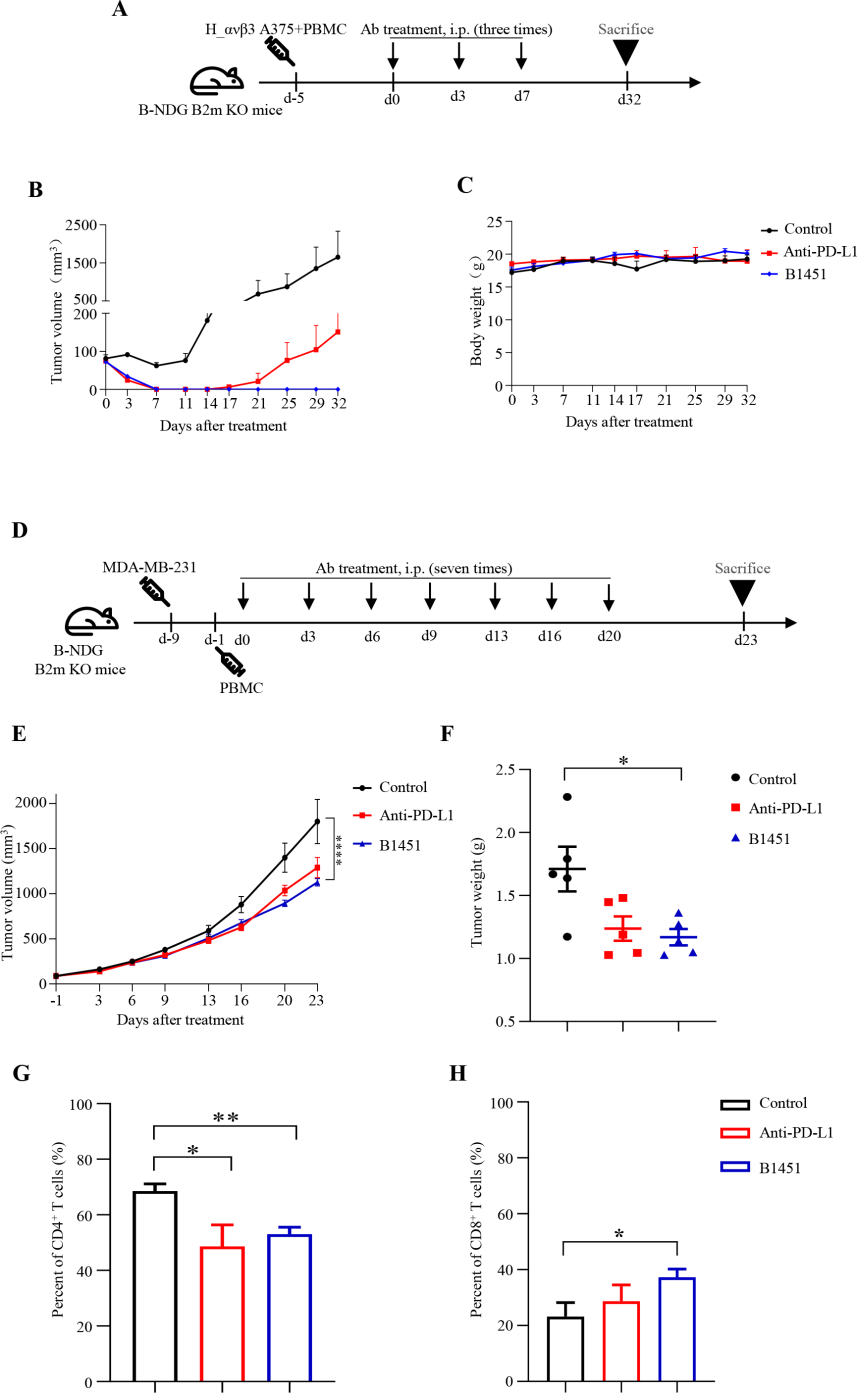
B1451 induces tumor regression in humanized mice *in vivo*. **(A)** Schematic representation of the H_αvβ3 A375 xenograft mouse model. B-NDG B2m KO immunodeficient mice were subcutaneously injected with a mixture of 1×10^6^ human PBMCs and 2×10^6^ H_αvβ3 A375 cells. When tumors sizes reached approximately 80 mm^3^, mice were randomly assigned to three groups (n=5). Test antibodies were administered intraperitoneally (i.p) on days 0, 3, 7, and the mice were sacrificed on day 32. Tumor volume **(B)**, and body weight **(C)** of mice are shown. **(D)** Schematic representation of the MDA-MB-231 xenograft mouse model. B-NDG B2m KO immunodeficient mice were subcutaneously injected with 1×10^7^ MDA-MB-231 cells. When tumors sizes reached 70–110 mm^3^, mice were randomized into three groups (n=5), and 5×10^6^ PBMCs were injected intraperitoneally. Test antibodies were administered intraperitoneally (i.p) on days 0, 3, 6, 9, 13, 16 and 20. Tumor volumes were measured twice per week, and the mice were sacrificed on day 23. Tumor volume **(E)**, tumor weight **(F)** of all mice are shown. The percentages of CD4^+^**(G)** and CD8^+^**(H)** T cells in MDA-MB-231 tumor tissues were analyzed by flow cytometry. Statistical significance was analyzed using ANOVA test and Unpaired t tests. ^*^p<0.05; ^**^p<0.01; ^****^p<0.0001.

To evaluate the tumor growth inhibitory potential of B1451, we established an H_αvβ3 A375 xenograft model in severely immunodeficient mice (Balb/c-Null for DNA Damage-Growth factor Attenuated Background, Beta-2 Microglobulin Knockout mouse, B-NDG B2m KO mice) mice (n=5). Five days after tumor implantation, mice received three doses of B1451 and anti-PD-L1 at 5 mg/kg. Treatment with B1451 reduced tumors to undetectable levels and resulted in stable, complete remission, whereas tumors in the anti-PD-L1 monotherapy group recurred by day 17 (**Figures 6A, B**). Importantly, B1451 treatment did not affect body weight (**Figure 6C**). To further confirm the therapeutic potential of B1451, we established an MDA-MB-231 xenograft model in B-NDG B2m KO mice (n=5). Treatment with anti-PD-L1 at a dose of 10 mg/kg modestly reduced tumor burden, whereas an equimolar dose of B1451 induced a significant reduction in tumor volume and tumor weight (**Figures 6D–F**).

Furthermore, flow cytometry analysis revealed a significant decrease in CD4^+^ T cells and an increase in CD8^+^ T cells within MDA-MB-231 tumor tissue in the B1451-treated group compared to the control group (**Figures 6G, H**), consistent with the results of multiplex immunofluorescence in MC38-hPD-L1 model.

**Figure 7:**
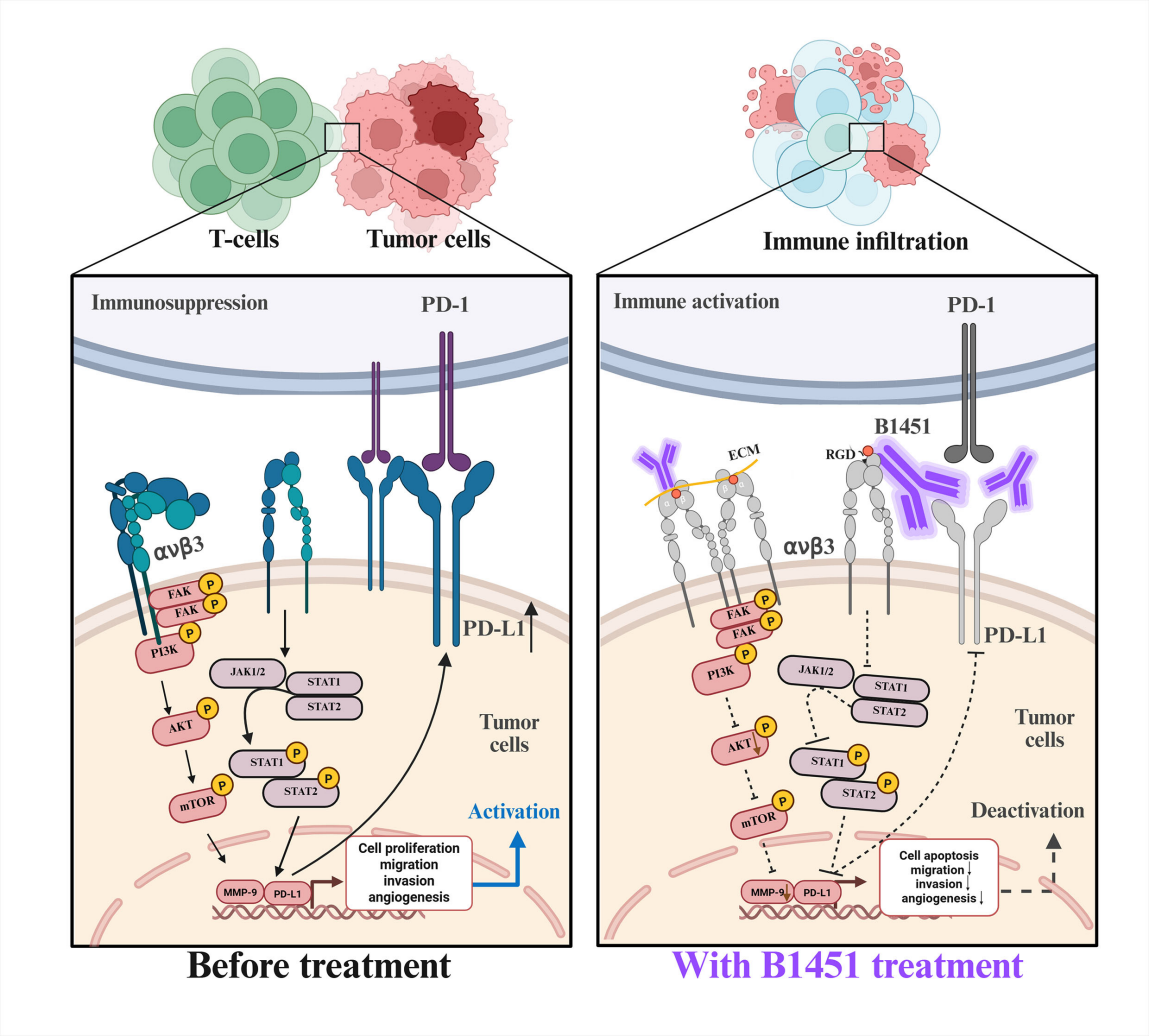
PD-L1 and αvβ3 are highly expressed in various human solid tumors, B1451, targeting both avβ3 and PD-L1, exhibits a stronger anti-tumor effect by increasing the infiltration of cytotoxic CD8^+^ T cells and reducing the microvessel density within tumors.

This study demonstrates that B1451 recognizes human PD-L1 and αvβ3 antigens, effectively blocking the PD-1/PD-L1 immune checkpoint and vitronectin/αvβ3 integrin interaction. B1451 inhibited tumor migration, adhesion and angiogenesis *in vitro* and induced superior anti-tumor effects *in vivo* compared to monotherapy. Mechanistically, the dual function antibody targeting avβ3 and PD-L1 enhances tumor growth suppression, through increased infiltration of cytotoxic CD8^+^ T cells and reduced intratumoral microvessel density (**Figure 7**). In summary, this research illustrates the ability of B1451, a dual function antibody targeting αvβ3 and PD-L1, to reverse immune evasion and exert synergistic anti-tumor effects. These results highlight the potential of B1451 as a promising strategy for immunotherapy in solid tumors, providing valuable insights for the development of combination therapies in the future.

### B1451 exhibits favorable pharmacokinetic and tolerance in mice

we investigated the pharmacokinetic properties of B1451 in C57BL/6N mice after a single intravenous injection of B1451 at 10 mg/kg and 100 mg/kg. The exposure of B1451 exhibits dose-dependency (online **Supplementary Table S2**), with C_max_ values of 131277.2 and 1498083.5 μg/L, and AUC_(0-t)_ values of 8574371.8 and 79724602.3 h·μg/L, respectively.

In terms of potential toxicity, we conducted a four-week repeated-dose toxicity study in C57BL/6N mice (a pharmacologically relevant species). C57BL/6N mice were administered of B1451 at 10 and 100 mg/kg once a week for a total of four doses (n=5/sex/group). The clinical signs, body weight, food consumption, organ weight and clinical chemistry were assessed. The results showed that in both dose groups, some animals exhibited prone posture and flushed ears starting from the second administration, which were considered to be caused by immunogenicity, and recovery was observed within 1 hour. There was no animal deaths and no abnormalities were found in body weight, food consumption, organ weight and clinical chemistry related to liver and kidney function (online **Supplementary Figure S5** and online **Supplementary Table S3**). B1451 was tolerated at all doses tested.

## Discussion

While blockade of the PD-1/PD-L1 axis has led to remarkable advances in cancer immunotherapy (31), the overall response rate among patients with advanced cancers remains low, largely due to immunosuppressive signaling pathways or insufficient immune cell infiltration (1). Emerging evidence suggests that integrin αvβ3, a critical player in tumor progression and metastasis, may contribute to immune evasion through regulation of PD-L1 expression (20). Preclinical studies have demonstrated that αvβ3 inhibitors can enhance the efficacy of anti-PD1 therapy and promote a stronger anti-tumor immune response (32). In the study, we demonstrated that both PD-L1 and αvβ3 are highly expressed in various human solid tumors, with αvβ3 being significantly overexpressed in patients classified as non-responders to anti-PD-L1 therapy. This finding highlights the potential of PD-L1 and αvβ3 as synergistic therapeutic targets for enhancing anti-tumor responses. However, no drugs targeting both pathways simultaneously have yet entered clinical trials.

Here, we describe the development of a novel PD-L1×αvβ3 bispecific antibody, B1451, that leverages a synergistic strategy. B1451 demonstrated high binding activity to both targets *in vitro* and displayed potent anti-tumor efficacy in various preclinical solid tumor models. These findings underscore the significant interplay between αvβ3 integrin and PD-L1 in regulating immune responses within the tumor microenvironment. Under pathological conditions, tumor cells overexpress αvβ3, and the ECM prote*in vitro*nectin promotes metastasis by binding to αvβ3 via its RGD domain and regulating the expression of MMPs through the PI3K signaling pathway (12, 17, 33). Consistent with these known mechanisms, we observed that B1451 inhibits tumor cell migration and invasion, which may be mediated by the downregulation of AKT phosphorylation and MMP9 expression. Moreover, previous research has shown that inhibition of αvβ3 reduces PD-L1 expression, which transforms cold tumors into immunologically hot tumors by promoting CD8^+^ T cell infiltration (20). In our study, B1451 demonstrated superior anti-tumor effect *in vivo* compared to anti-PD-L1 monotherapy by increasing CD8^+^ T cell infiltration into tumor tissues. These results suggest that dual targeting strategies, aimed at modulating multiple pathways, could significantly improve cancer treatment outcomes (34). This research not only deepens our understanding of the molecular mechanisms underlying tumor progression and immune evasion but also opens the door for the development of novel bispecific antibody-based therapies targeting PD-L1 and αvβ3. These preclinical findings highlight the therapeutic promise of B1451, offering exciting opportunities for future clinical application in cancer immunotherapy.

It is also worth noting that BsAb have emerged as a key class of therapeutic agents in cancer treatment. By targeting dual tumor-associated antigens, BsAb increase tumor selectivity while simultaneously modulating multiple functional pathways, making them an attractive strategy for enhancing therapeutic efficacy (29, 35). While limitations of this study include the lack of clinical validation, B1451 exhibits promising anti-tumor effects in preclinical models, paving the way for further translational studies.

In summary, our study introduces a novel bispecific antibody, B1451, which fuses an integrin αvβ3 binding peptide to the C-terminal heavy chain of the anti-PD-L1 monoclonal antibody Atezolizumab via a (G4S)×3 linker. This BsAb demonstrated robust anti-tumor activity by promoting CD8^+^ T cell infiltration and inhibiting angiogenesis in preclinical models. Although further clinical validation is required, these findings represent a promising step toward the development of more effective, targeted cancer therapies, providing new opportunities for advancing cancer immunotherapy.

## Materials and methods

### Database analysis

The molecular profiles of patients receiving immunotherapy and the relationship between αvβ3 expression and PD-L1 infiltration levels in hepatocellular carcinoma, pancreatic cancer, renal cancer, and urothelial cancer were analyzed using the Xiantao tool (https://www.xiantao.love/). Additionally, the correlation between αvβ3 expression and immunotherapy response was evaluated using the TISIDB database (http://cis.hku.hk/TISIDB) (36).

### Generation of B1451

B1451 was constructed as a symmetric PD-L1×αvβ3 bispecific antibody. To achieve this, an integrin αvβ3-binding peptide was conjugated to the C-terminal of the heavy chain of the anti-PD-L1 monoclonal antibody Atezolizumab using a (G4S)×3 linker. The variable region sequences of the Atezolizumab antibody are derived from SEQ ID NO:20 and SEQ ID NO:21, as disclosed in Chinese patent CN200980149532. The sequence of the integrin αvβ3-binding peptide was obtained from SEQ ID NO:4, as disclosed in Chinese patent CN202111581908.

### Binding affinity detection by surface plasmon resonance

The binding affinities of B1451 for PD-L1 and αvβ3 antigens were measured using a Biacore T200 (GE healthcare, USA). For PD-L1 affinity detection, anti-human IgG (Fc) was immobilized onto a CM5 sensor chip using the Human Antibody Capture Kit (Cytiva, Cat# BR100839), and B1451 was captured on the sensor surface at approximately 35 RU. Subsequently, 2-fold serial dilutions of human PD-L1 (concentration range: 0.78125–50 nM) was injected over the sensor chip with an association time of 1500 s. Surface regeneration was carried out using a pulse injection of 10 mM glycine-HCl (pH 1.5). For αvβ3 affinity detection, B1451 was similarly captured on an anti-human IgG (Fc)-pre-immobilized CM5 sensor chip at approximately 35 RU. Serial 2-fold dilutions of human αvβ3 (concentration range: 12.5–400 nM) were injected over the sensor chip with an association time of 120 s. Surface regeneration was performed using a pulse injection of 10 mM glycine-HCl (pH 1.5). The binding affinities of B1451 for both antigens were determined by fitting the data to a 1:1 binding model using Biacore T200 Evaluation Software (Version 3.2.1).

### Cell lines and cell culture

The U251, SU.86.86, ACHN cells lines were obtained from Pricella, while the MDA-MB-468, MDA-MB-231, H22, and B16/F10 cell lines were acquired from Cobioer. The human PD-L1-expressing MC38 cell line (MC38-hPD-L1) was obtained from Shanghai Model Organisms Center, Inc., The human αvβ3 high-expressing A375 cell line (H_αvβ3 A375) was established by Genomeditech. Human PD-1-expressing NFAT-luc reporter Jurkat cells and CHO cells expressing both PD-L1 and CD3scFv (CHO-PD-L1-CD3L) were purchased from National Institutes for Food and Drug Control. Frozen human peripheral blood mononuclear cells (PBMCs) were purchased from ORiCELLS. All cell lines were cultured under standard conditions at 37 °C in a humidified atmosphere with 5% CO_2_, using appropriate standard growth media.

### Flow cytometry

To assess PD-L1 and αvβ3 expression in tumor cells, cell suspensions were harvested and counted. A total of 1×10^5^ cells were stained with either anti-PD-L1 antibody (Atezolizumab, Roche, USA, Lot# H0211B01) or APC-conjugated anti-human CD51/61 antibody (Biolegend, USA, 304416). The samples wewe analysed using a NovoCyte Quanteon flow cytometer (Agilent, USA), and data were processed using the NovoExpress software.

For tumor microenvironment immune infiltration analysis, MDA-MB-231 tumors were harvested and homogenized to create single-cell suspensions. Cells were subsequently stained with the following antibodies: APC anti-human CD45 (Biolegend, USA, Cat# 982304), APC_Fire 750 anti-human CD8a Antibody (Biolegend, USA, Cat# 344746) and Pacific Blue anti-human CD4 Antibody (Biolegend, USA, Cat# 317424). Analyses were conducted on the NovoCyte Quanteon flow cytometer(Agilent, USA), and data were processed using NovoExpress software.

### Cell binding assay

The binding affinities of B1451 for cells were evaluated using flow cytometry. CHO-PD-L1-CD3L pre-blocked for αvβ3, H_αvβ3 A375 pre-blocked for PD-L1, and MDA-MB-468 cells were used to detect the binding specificity of B1451. MDA-MB-231 and U251 cells, which co-express both PD-L1 and avβ3, was selected to evaluate the binding activity of B1451. To detect the binding activity of B1451 to αvβ3 antigen, H_αvβ3 A375 cells were pre-treated with 10 μg/mL anti-PD-L1 to occupy the PD-L1 antigen, and then stained with serial dilutions of PE-labeled B1451 or reference reagents for 1 h at 4°C. To detect the binding of B1451 to PD-L1 antigen, CHO-PD-L1-CD3L cells were pre-treated with 10 μg/mL HM-3 to occupy the αvβ3 antigen, and then stained with serial dilutions of PE-labeled B1451 or reference reagents for 1 h at 4°C. Then, the geometric mean fluorescence intensity (MFI) was recorded using NovoCyte Quanteon flow cytometer(Agilent, USA). To detect the binding of B1451 with other cells, we adopt a conventional method. Briefly, tumor cells were seeded in 96-well V-Bottom plates (Corning, USA, Cat# 3897) and incubated with serial dilutions of B1451 or reference reagent for 1 h at 4°C. After incubation, the plates were washed twice with cold DPBS and subsequently stained with PE-conjugated anti-human IgG Fc (Biolegend, USA, Cat# 409304) for 30 min at room temperature in the dark. Following staining, the cells were washed three times with cold DPBS, resuspended in DPBS, and analyzed on a NovoCyte Quanteon flow cytometer(Agilent, USA). The geometric mean fluorescence intensity (MFI) was recorded to determine binding activity.

### PD-1/PD-L1 blockade assay

CHO-PD-L1-CD3L cells were seeded at a density of 5×10^4^ cells per well in 96-well white plates (Corning, USA, Cat# 3610) and incubated overnight at 37°C in a humidified incubator with 5% CO_2_. The following day, PD-1 effector cells were added at a density of 1×10^5^ cells per well. Serial dilutions of B1451 or reference reagents were then incubated with the co-cultured cells for 6 hours at 37°C in a CO_2_ incubator. After the 6-hour incubation, an equal volume of Bio-Lite Luciferase Assay System reagent (Vazyme, Cat# DD1201-02) was added to each well. Luminescence was measured using the Infinite 200 PRO microplate reader (TECAN, Switzerland) to analyze luciferase activity.

### αvβ3/vitronectin blockade assay

The human vitronectin (sigma, USA, Cat# SRP3186-250UG) was immobilized on 96-well plates at a concentration of 2 μg/mL by incubating overnight at 4 °C. The following day, the liquid was discard, and the wells was washed four times with washing buffer. The plates were then blocked with 5% BSA in washing buffer for 1.5 h at 37°C. After blocking, the wells were washed four more times with washing buffer. Subsequently, ITGAV&ITGB3 heterodimer protein (Acro, USA, Cat# IT3-H52E3, 2.5 μg/mL) and serially diluted B1451 or reference reagents solution (1.5625-800 μg/mL) were added to the wells and incubated at room temperature for 1.5 h. After incubation, purified mouse antibody CD51/CD61 (R&D, USA, Cat#555504) was incubated for 1 h at 37°C as the primary antibody. Anti-mouse IgG (whole molecule)-peroxidase antibody produced in sheep (Sigma, USA, Cat# A5906) was incubated for 1 h at 37 °C as the secondary antibody. Finally, tetramethylbenzidine (TMB) substrate solution was added for color development.

### Cell migration assay

For cell migration experiments, 1×10^4^ H_αvβ3 A375 cells per well were seeded into the upper compartments of 24-well Transwell culture chambers (8.0 μm pore size, Corning, USA, Cat# 3422), with or without serial dilutions of B1451 or fixed concentration of HM-3 and anti-PD-L1. The lower chambers were filled with 400 μL of medium containing 2% FBS. All experimental groups were conducted in triplicate. Cells were incubated at 37°C in a humidified atmosphere with 5% CO_2_ for 14 h. After incubation, cells that migrated to the lower surface of the membrane was fixed with 4% formaldehyde and stained with crystal violet for 10 min. Images were captured, and the number of cells on the lower surface of each insert was then counted.

### Cell invasion assay

For cell invasion experiments, 8×10^3^ H_αvβ3 A375 cells were resuspended in 100 μL of serum-reduced medium (1% FBS) and seeded into Matrigel-precoated Transwell inserts (Corning, USA, Cat# 354234), with or without serial dilutions of B1451 or fixed concentration of HM-3 and anti-PD-L1. The lower chambers contained 400 μL of complete medium supplemented with 10% FBS. All treatment groups were performed in triplicate. Cells were incubated at 37°C in a humidified atmosphere with 5% CO_2_ for 24 h. After incubation, cells that invaded and adhered to the lower surface of the membrane was fixed with 4% formaldehyde and stained with crystal violet for 10 min. Images were captured, and the number of cells on the lower surface of each insert was counted.

### Tube formation

For tube formation experiments, 96-well plates were pre-coated with 100 μL/well of Matrigel^®^ Matrix (Corning, USA, Cat# 354234) and incubated at 37°C until the matrix solidified completely. HUVECs were seeded onto the gel at a density of 6×10^5^ cells/well in the presence or absence of test articles. All experimental groups were performed in triplicate. Cells were incubated at 37°C in a humidified atmosphere with 5% CO_2_ for 18 h. Images of tube-like structures were captured using microscope, and the total tube length formed in each well was quantified using Image J software.

### Cell apoptosis assay

For apoptosis analysis, H_αvβ3 A375 cells were cultured in 6-well plate and treated with B1451 at concentrations of 20 or 100 μg/mL for 24 h. After treatment, cells were harvested, washed twice with ice-cold PBS and stained with Annexin V-FITC/PI (Vazyme, Cat# A211) according to the manufacturer’s protocol. Samples were analyzed using a NovoCyte Quanteon flow cytometer (Agilent, USA), and the data were processed with NovoExpress software.

### Immunoblot assay

2×10^6^ H_αvβ3 A375 cells in 2 mL were seeded into 6-well plates and incubated with serially diluted B1451 (10, 40 and 160 μg/mL) for 24 h. After that, cells were harvested and lysed with cell lysis buffer (CST, USA, Cat# 9803), followed by rotating and centrifuging to collect the supernatant. Proteins were sepatated using SDS-PAGE and transferred to polyvinylidene difluoride (PVDF) membranes. The membrane was incubated with the primary antibodies overnight at 4°C and incubated with the secondary antibodies for 1 h at room temperature. Protein expression was measured using Omni-EC™(epizyme), and the emitted signals were imaged using Tanon 4800 Multi gel image analysis system (Tanon). The following primary antibodies were used as follows: rabbit anti-MMP-9 polyclonal antibody (Proteintech, USA, Cat# 10375-2-AP), mouse anti-AKT monoclonal antibody (Proteintech, USA, Cat# 60203-2-Ig), rabbit anti-phospho-AKT (Ser473) antibody (CST, USA, Cat# 4060S) and mouse anti-GAPDH monoclonal antibody (Proteintech, USA, Cat# 60004-1-Ig)). The secondary antibodies used were as follows: Anti-rabbit IgG HRP-linked (CST, USA, Cat# 7074) and Anti-mouse IgG HRP-linked (CST, USA, Cat# 7076).

### Immunohistochemical analysis

Immunohistochemistry staining for PD-L1 and αvβ3 integrin was performed on paraffin-embedded sections of human pancreatic cancer, renal cancer, and urothelial cancer tissues, as well as liver cancer microarray samples (20221016R30) by Histopath, Inc. Staining was carried out using a commercial rabbit anti-PD-L1 antibody (Abcam, Cat# ab205921) and a mouse anti-Integrin alpha V beta 3 antibody (Abcam, Cat# ab7166). Immunohistochemical procedures were conducted as previously described (37).

Staining frequency was categorized into four groups:

0: No or < 10 minimally labeled cells.
1: <10% of cells.
2: < 50% of cells.
3: <90% of cells.
4: Essentially all cells.

Staining intensity was also graded into three groups:

0: No or < 10 minimally labeled cells.
1: Low.
2: Medium.
3: High.

For CD31 staining, 3 μm tissue sections of MC38-hPD-L1 tumors were prepared using the Leica RM2255 rotary microtomes and baked for 1 hour using Leica HT1220 slide warmer before staining. Tissue sections were stained for mouse CD31 using a commercial rabbit anti-CD31 antibody (CST, USA, Cat#77699) as described previously (37). Images were were captured using Leica DFC7000T CCD and the density of CD31-positive blood vessels was quantified using StrataQuest software(Ver. 7.1.1.119).

### Multiplex immunofluorescence staining

mIF was performed as previously described (38). Briefly, MC38-hPD-L1 tumor tissue sections were pre-treated in the same way as for CD31 staining. A 3-plex panel was used with the following antibodies: anti-CD4 (CST, USA, Cat# 25229), anti-CD8 (CST, USA, Cat# 98941) and anti-PD-L1 (CST, USA, Cat# 13684) were used as the primary antibody at a final dilution of 1:200. Anti-rabbit HRP (CST, USA, Cat# 8114) was utilized as the secondary antibody. Alexa Fluor™ fluorophores were applied to visualize biomarkers: Alexa Fluor™ 555 (CD4, Invitrogen, Cat# B40955), Alexa Fluor™ 594 (CD8, Invitrogen, Cat# B40957), Alexa Fluor™ 488 (PD-L1, Invitrogen, Cat# B40953). Tissue slides were counterstained with DAPI and mounted with prolong gold antifade reagent (CST, USA, Cat# 9071). Whole-slides imaging was performed at ×10 magnification using the Leica DFC7000T) for gross visualization. Representative regions were scanned at 20× magnification for quantitative analysis.

### Xenograft model

Suspensions of human or mouse tumor cells were inoculated subcutaneously into specific pathogen-free female mice. When tumors reached an appropriate volume, tumor-bearing mice were randomized into treatment and vehicle control groups based on tumor volumes and body weight, and dosing was initiated (designated as Day 0). Each substance was administered intraperitoneally twice per week. Tumor volumes were measured using calipers two or three times per week and calculated using the formula: tumor volume=(length×width^2^)/2. Tumor growth inhibition (TGI, %) was calculated for each group using the following formula: TGI (%) = [1-(Ti-T0)/(Vi-V0)] ×100 (%), where Ti is the average tumor volume of a treatment group on a given day, T0 is the average tumor volume of the treatment group on the first day of treatment, Vi is the average tumor volume of the vehicle control group on the same day with Ti, and V0 is the average tumor volume of the vehicle control group on the first day of treatment.

### Pharmacokinetics study

Male and Female C57BL/6N mice were randomly assigned to two experimental groups (5/sex/group). A single 10 mg/kg and 100 mg/kg intravenous bolus of B1451 were administered. Blood samples were collected from the posterior orbital venous plexus at predetermined time points and subsequently analyzed using ELISA. Human PD-L1 protein (hFc-tag) (ACRO, USA, Cat# PD-1-H5258) was used as coated antigen. SC1483A-customized monoclone antibody (GenScript, CN, Cat# C8499DC070-1) and HRP-conjugated affinipure goat anti-mouse IgG (H+L) (Proteintech, USA, Cat# SA00001-1) were used as detection antibodies.

### Four-week repeated-dose toxicity study

Male and Female C57BL/6N mice were randomly allocated into control and treatment groups. The treatment group received B1451 at 10 and 100 mg/kg once a week for a total of four doses. Body weights and food consumption were recorded once or twice a week. At the experimental endpoint, these animals were humanely euthanized. Subsequently, serum was collected to measure clinical chemistry parameters, and major organs (heart, liver, spleen, lungs, kidneys, and reproductive organs) were harvested and weighed.

### Statistical analysis

All data were graphed using GraphPad Prism software and the results of statistical analyses are reported in the figure legends where applicable as follows: ^*^P < 0.05, ^**^P < 0.01, ^***^P < 0.001, ^****^P < 0.0001, ns, not significant.

## Data Availability

The original contributions presented in the study are included in the article/**Supplementary Material**. Further inquiries can be directed to the corresponding authors.
