# Supplementary material for: Dual function antibody targeting αvβ3 and PD-L1 provide a promising strategy for solid tumor therapy
Source: Front Immunol. 2026 Jan 12;16:1691774. doi: 10.3389/fimmu.2025.1691774 (PMC12833063; doi:10.3389/fimmu.2025.1691774)
Supplement: Supplementary file 1 [file DataSheet1.pdf]

Supplementary Figure S1

A

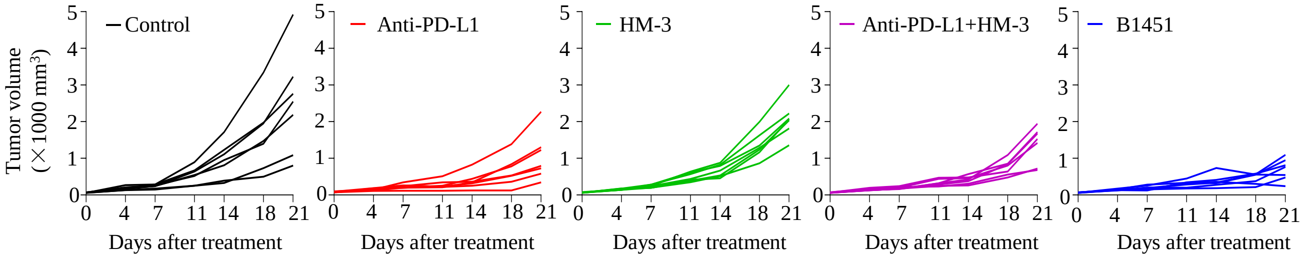

B

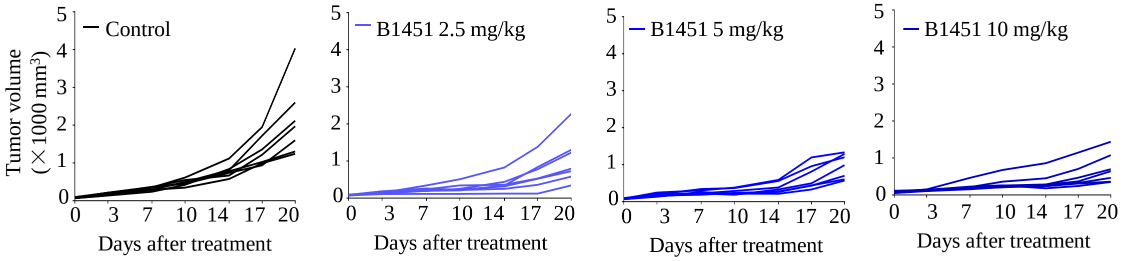

Supplementary Figure S1. Tumor growth curves of all mice are shown.

Supplementary Figure S2

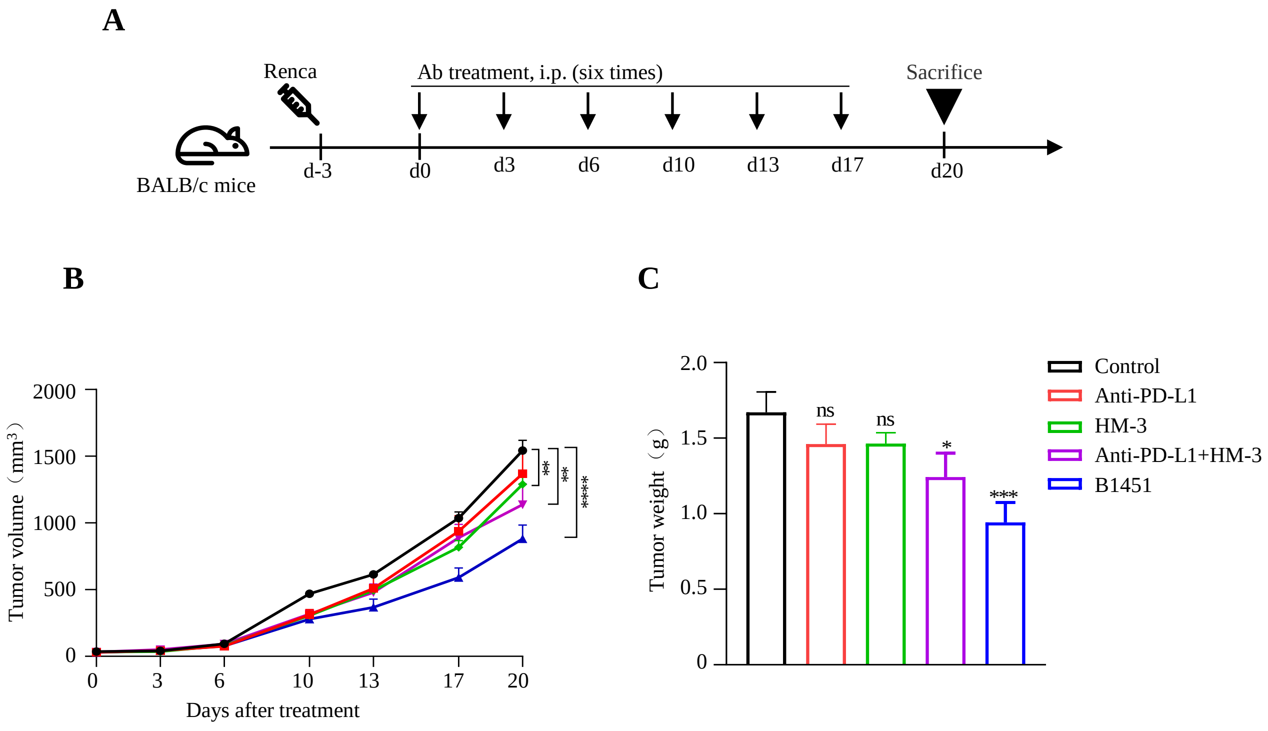

Supplementary Figure S2. B1451 reduces tumor burden in a xenogeneic tumor model of Renca. (A) Schematic representation of the xenograft mouse model. BALB/c mice were subcutaneously injected with 5×10^5 Renca cells. 3 days after tumor cell injection. tumor-bearing mice were randomly divided into different groups and treated with the indicated antibodies six times (n=6). Tumor volumes were measured twice per week, and mice were sacrificed on day 20. (B-C) Tumor growth curves and tumor weight of all mice are shown. **p<0.01; ***p<0.001; ****p<0.0001; ns, not significant.

Supplementary Figure S3

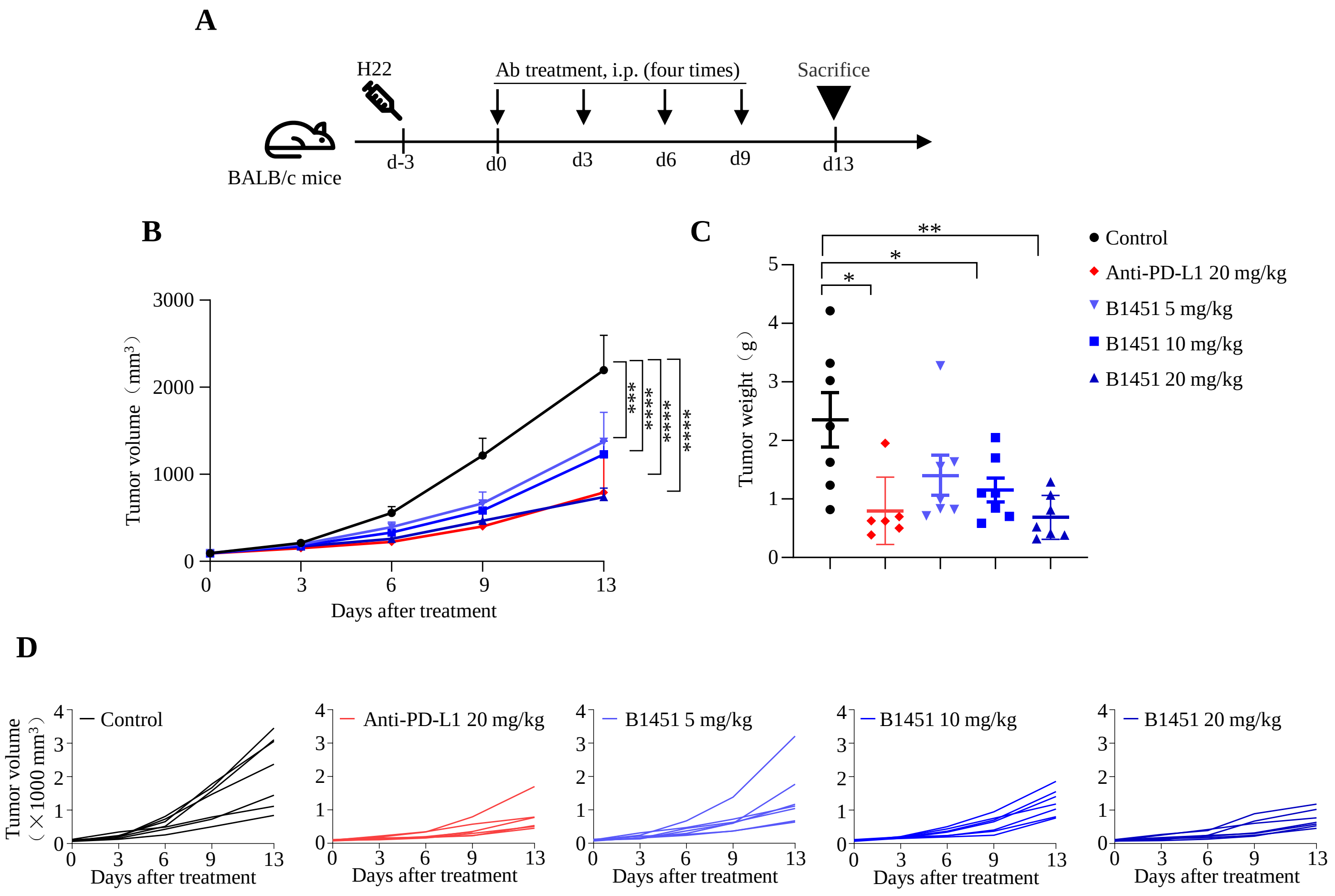

Supplementary Figure S3. B1451 reduces tumor burden in a xenogeneic tumor model of H22. (A) Schematic representation of the xenograft mouse model. BALB/c mice were subcutaneously injected with 1×10^6 H22 cells. 5 days after tumor cell injection, when the mean tumor size reached approximately 94 mm^3, tumor-bearing mice were randomly divided into different groups and treated with the indicated antibodies four times (n=7, n=6 [the group of Anti-PD-L1]). Tumor volumes were measured twice per week, and mice were sacrificed on day 13. (B-D) Tumor growth curves and tumor weight of all mice are shown. $^{*}p<0.05$; $^{**}p<0.01$; $^{***}p<0.001$; $^{****}p<0.0001$.

Supplementary Figure S4

A

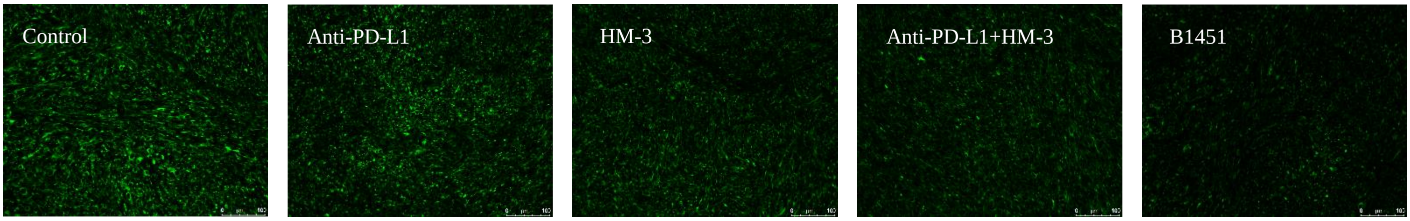

B

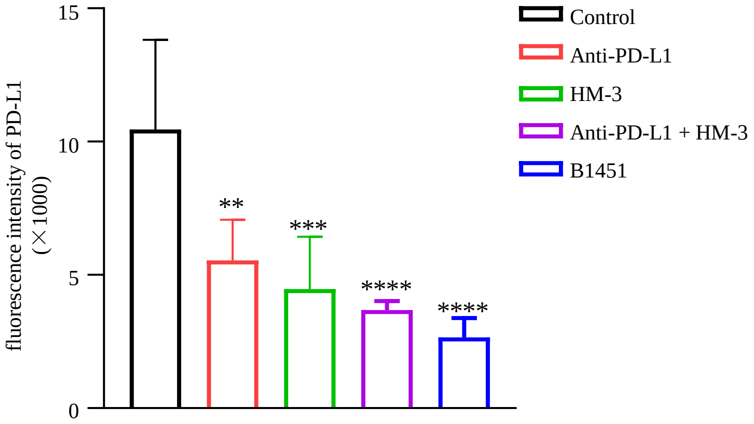

Supplementary Figure S4. (A-B) Representative immunohistochemistry images evaluating PD-L1 expression, with quantification of the fluorescence intensity of PD-L1 among all tissues cells performed manually. Statistical significance was analyzed using ANOVA test. $**p<0.01$; $***p<0.001$; $****p<0.0001$.

Supplementary Figure S5

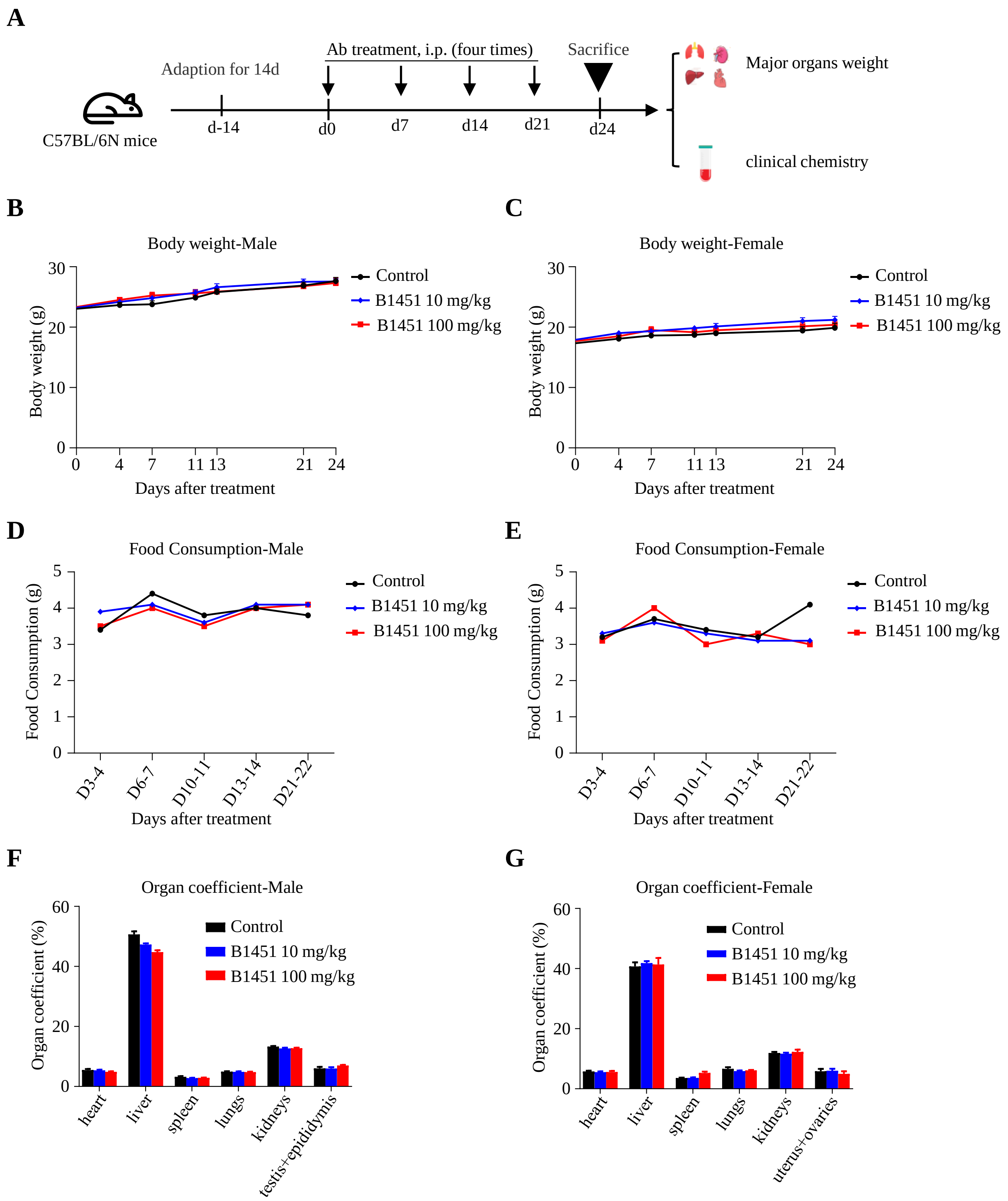

Supplementary Figure S5. Four-week repeated-dose toxicity study of B1451 in mice. (A) Schematic representation of four-week repeated-dose toxicity study. (B-C) The effect of B1451 on body weight in mice. (D-E) The effect of B1451 on food consumption in mice. (F-G) The effect of B1451 on organ coefficient in mice.
