# Supplementary material for: Dual function antibody targeting αvβ3 and PD-L1 provide a promising strategy for solid tumor therapy
Source: Front Immunol. 2026 Jan 12;16:1691774. doi: 10.3389/fimmu.2025.1691774 (PMC12833063; doi:10.3389/fimmu.2025.1691774)
Supplement: Supplementary file 2 [file DataSheet2.pdf]

Supplemental Table S1. Scoring statistics of immunohistochemistry staining frequency and intensity for PD-L1 and αvβ3 are shown across various tumors

Histology	Number of Cores	PD-L1									αvβ3								
		Frequency					Intensity				Frequency					Intensity			
		0	1	2	3	4	0	1	2	3	0	1	2	3	4	0	1	2	3
Urothelial carcinoma	30	0/30	4/30	18/30	7/30	1/30	0/30	18/30	12/30	0/30	3/30	5/30	19/30	2/30	1/30	3/30	16/30	9/30	2/30
Renal carcinoma	50	13/50	11/50	22/50	3/50	1/50	13/50	7/50	21/50	9/50	7/50	18/50	22/50	2/50	1/50	7/50	10/50	17/50	16/50
Pancreatic cancer	30	7/30	3/30	10/30	10/30	0/30	7/30	19/30	4/30	0/30	3/30	5/30	15/30	7/30	0/30	3/30	24/30	3/30	0/30
Hepatocellular carcinoma	40	3/40	16/40	14/40	5/40	2/40	3/40	31/40	6/40	0/40	24/40	13/40	2/40	1/40	0/40	24/40	15/40	1/40	0/40
Scoring Criteria		Staining Frequency:<br>0= No labeled cells<br>1=<10% of cells<br>2=<50% of cells<br>3=<90% of cells<br>4=essentially all cells					Staining Intensity:<br>0=No labeled cells<br>1=Low<br>2=Medium<br>3=High				Staining Frequency:<br>0= No labeled cells<br>1=<10% of cells<br>2=<50% of cells<br>3=<90% of cells<br>4=essentially all cells					Staining Intensity:<br>0=No labeled cells<br>1=Low<br>2=Medium<br>3=High			

Supplemental Table S2 Pharmacokinetic parameters of B1451 in mice

Dose (mg/kg)	Sex	T_{max} (h)	$T_{1/2}$ (h)	C_{max} (μg/L)	AUC_{0-t} (h·μg/L)
10	M	3	51±28	145225.3±6157.2	9344847.7±565492.6
	F	3	91±25	117329.1±12188.3	7803895.9±701598.1
100	M	3	135±52	1562016.0±54092.2	85126266.6±3578037.2
	F	3	109±37	1434151.0±126087.1	74322937.9±6613700.0

Supplemental Table S3 Clinical chemistry parameters of B1451 in mice

Sex	Dose (mg/kg)	No#	ALT (U/L)	AST (U/L)	ALP (U/L)	ALB (g/L)	UA (μmol/L)	UREA (mmol/L)	CREA (mmol/L)	TG (mmol/L)	CHO (mmol/L)
Male	0	1	57.6	170.4	74	16.54	118	8.32	5	0.54	2.55
		2	70.2	191.7	157	17	96	13.39	8	1.09	2.13
		3	69.5	248.8	118	15.26	118	7.38	5	0.52	2.04
		4	59.3	172.3	94	16.33	111	8	4	0.5	2.02
		5	113.2	275.7	70	15.49	124	10.15	6	0.58	1.97
	10	1	41.7	78.5	108	17.96	112	9.08	7	1.01	2.86
		2	37.7	76	100	16.51	121	8.04	6	0.64	2.27
		3	58	110.5	99	20.1	151	9.06	6	0.73	2.64
		4	96	120.5	109	20.1	148	6.83	5	0.72	2.63
		5	39.4	84.9	110	18.45	126	6.37	5	0.69	2.58
	100	1	31.1	68.5	88	17.15	84	7.27	7	0.63	2.51
		2	43.3	99.7	86	15.49	88	4.99	7	0.7	2.72
		3	57.5	95.9	87	16.74	118	6.11	4	0.61	2.4
		4	30.2	112.5	115	21.55	126	8.21	5	0.66	2.64
		5	37.2	108.8	103	20.65	140	7.55	4	0.7	2.78
Female	0	1	32.7	110.5	134	19.64	92	6.89	4	0.6	1.27
		3	30.9	130.4	160	21.35	71	7.79	6	0.63	2.1
		4	28.3	103.4	139	19.96	101	10.21	8	0.52	2.13
		5	32.9	95.5	176	24.76	152	11.77	8	0.58	2.27
		5	29.8	115.8	163	23.72	152	9.12	6	0.8	2.36
	10	1	30.9	101.5	154	23.08	92	5.84	7	0.6	2.23
		2	27	102.5	148	21.9	81	6.38	4	0.7	2.4
		3	32.7	117.8	145	21.06	69	7.22	5	0.71	2
		4	34.7	98.2	145	22.53	63	6.92	6	0.84	2.62
		5	34.3	114.9	177	21.58	61	7.08	4	0.65	1.8
	100	1	32.3	134.6	168	22.59	79	9.49	8	0.68	2.42
		2	48	154.6	166	23.6	80	9.62	7	0.68	2.74
		3	31.8	117.6	149	21.61	85	9.08	6	0.58	1.92
		4	24.4	94.3	151	18.33	81	7.37	7	0.39	1.19
		6	43.8	108.4	174	20.8	84	11.95	7	0.58	2.09
